# Comparison of percutaneous endoscopic and open posterior lumbar interbody fusion for the treatment of single-segmental lumbar degenerative diseases

**DOI:** 10.1186/s12891-022-05287-9

**Published:** 2022-04-07

**Authors:** Li-Ming He, Kuo-Tai Chen, Chien-Min Chen, Qiang Chang, Lin Sun, Yan-Nan Zhang, Jian-Jun Chang, Hao-Yu Feng

**Affiliations:** 1 Department of Orthopaedic Surgery, Shanxi Bethune Hospital(Shanxi Academy of Medical Sciences), China. (No. 99, Longcheng street, Taiyuan, Shanxi Province China; 2 Department of Neurosurgery, Chang Gung Memorial Hospital Chiayi Branch, Chiayi Branch, Chiayi, Taiwan, (No. 6, W. sec., Jiapu Rd., Puzi City, 12, Chiayi, Taiwan 613016; 3 Division of Neurosurgery, Department of Surgery, Changhua Christian Hospital, No. 135 Nanxiao St., Changhua City, Changhua County 500, Taiwan, Changhua, Taiwan; 4 School of Medicine, Kaohsiung Medical University, Taiwan (No. 100, Shih-Chuan 1st Road, Sanmin Dist Kaohsiung City, Taiwan 80708; 5 College of Nursing and Health Sciences, Dayeh University, Taiwan (No. 168, University Rd, Dacun, Changhua Taiwan 515006

**Keywords:** Posterior lumbar interbody fusion, Endoscope, Minimally invasive, Lumbar degenerative diseases

## Abstract

**Background:**

Endoscopic lumbar interbody fusion has become an emerging technique. Some researchers have reported the technique of percutaneous endoscopic transforaminal lumbar interbody fusion. We propose percutaneous endoscopic posterior lumbar interbody fusion (PE-PLIF) as an alternative approach. The purpose of this study was to assess the clinical efficacy of PE-PLIF by comparing percutaneous endoscopic and open posterior lumbar interbody fusion (PLIF).

**Methods:**

Thirty patients were enrolled in each group. Demographic data, perioperative data, and radiological parameters were collected prospectively. The clinical outcomes were evaluated by visual analog scale (VAS) and Oswestry Disability Index (ODI) scores.

**Results:**

The background data were comparable between the two groups. The mean operation time was longer in the PE-PLIF group. The PE-PLIF group showed benefits in less blood loss and shorter hospital stay. VAS and ODI scores significantly improved in both groups. However, the VAS score of low-back pain was lower in the PE-PLIF group. The satisfaction rate was 96.7% in both groups. The radiological outcomes were similar in both groups. In the PE-PLIF group, the fusion rate was 93.3%, and the cage subsidence rate was 6.7%; in the open PLIF group, the fusion and cage subsidence rates were 96.7% and 16.7%. There were minor complications in one patient in the PE-PLIF group and two in the open PLIF group.

**Conclusions:**

The current study revealed that PE-PLIF is safe and effective compared with open PLIF. In addition, this minimally invasive technique may enhance postoperative recovery by reducing tissue damage and blood loss.

## Introduction

Trial and error are common features in the progress of any surgical technique. In the 1970s, Hijikata from Japan [1] first performed posterolateral lumbar nucleotomy. In the 1980s, Parviz Kambin [2, 3] further improved the technique and proposed the “Kambin triangle.” Schreiber [4] first used endoscopic techniques in posterolateral lumbar nucleotomy. Around 2000, Tony Yeung [5] and Hal Mathews [6] further improved percutaneous endoscopic lumbar nucleotomy. In early 2000, Sebastian Rutten [7], a spine surgeon in Germany, improved and used a translaminar endoscopic technique. With the advancement of surgical techniques and instruments, the indications for endoscopic nucleotomy are expanding, and this procedure has satisfactory clinical efficacy, making it applicable to various types of disc herniation [8–10]. However, the initial endoscopic operation is not intended for lumbar instability, lumbar spondylolisthesis, or severe intervertebral stenosis, all of which require lumbar interbody fusion to restore lumbar stability and disc height. There have been several minimally invasive lumbar interbody fusion techniques developed using different tubular retractors, such as minimally invasive transforaminal lumbar interbody fusion (MI-TLIF) and oblique lumbar interbody fusion. These retractor systems may still cause certain collateral tissue damage. In addition, lumbar interbody fusion through tubular retractors has limited visualization and a steep learning curve.

Endoscopic lumbar interbody fusion (Endo-LIF) has become an emerging technique in minimally invasive spinal surgery. Morgenstern R et al. [11] first reported the use of endoscopic interbody fusion to treat severe intervertebral stenosis. During the operation, they removed the ventral portion of the superior articular process to expand the intervertebral foramen and then used a B-twin expandable cage for interbody fusion, with satisfactory clinical efficacy. Endoscopic fusion techniques are evolving with the continuous improvement in surgical instruments and cages. Some researchers have reported the technique of percutaneous endoscopic transforaminal lumbar interbody fusion. We proposed PE-PLIF as an alternative for Endo-LIF. The surgical approach of PE-PLIF is located on one side of the articular process and the lamina. This approach can obtain a local view of the open PLIF operation, and the positions of the unilateral nerve root decompression and cage placement are similar. PE-PLIF is more precise and less traumatic than open PLIF. In this study, we compared PE-PLIF and open PLIF to investigate the feasibility and effectiveness of PE-PLIF.

## Materials and methods

### Study design

This study was approved by the Ethics Committee of Shanxi Bethune Hospital. Patient consent was obtained before the study. All operations were performed by the same surgical team. The enrolled cases in this study started after approximately 30 operations had been completed and the surgical procedures were relatively stable. Thirty patients undergoing single-segmental PE-PLIF at our hospital between June 2019 and October 2019 were enrolled in this study. Moreover, 30 patients undergoing single-segment open PLIF were matched for comparison.

Inclusion criteria: All patients who reported sustained chronic lower back pain with or without radiation to the lower legs due to the following diagnosis were included: (1) single-segmental lumbar degenerative disease, including lumbar disc herniation with lumbar instability, lumbar disc herniation with lumbar spondylolisthesis (Meyerding grade I), and lumbar disc herniation with severe intervertebral stenosis; and (2) lack of efficacy after ≥ 3 months of conservative treatment or worsening symptoms. We excluded patients with (1) Far-lateral lumbar disc herniation; (2) spinal deformity, old fractures, or ankylosing spondylitis. Lumbar instability was defined as the X-ray of lumbar hyperflexion and hyperextension showing a change in the intervertebral angle greater than 10° or back-and-forth sliding of the vertebral trailing edge of 3 mm or more. Severe intervertebral stenosis was defined as a disc height of 6 mm or less with lumbar outlet compression symptoms.

### Demographic data

The age of the patients in the PE-PLIF group was 51.6 ± 13.4 years (range 32–78 years). Sixteen patients underwent PE-PLIF at the L4-5 segment, and another 14 patients at the L5-S1 segment. The open PLIF group patients were aged 55.9 ± 14.3 years (range 32–79 years). Eight patients underwent open PLIF at the L4-5 segment, and 22 patients at the L5-S1 segment. Both groups were composed of 18 male and 12 female patients. The mean follow-up time was 24.7 ± 1.1 months (range 22–26 months) in the PE-PLIF group and 25.3 ± 1.5 months (range 22–28 months) in the open PLIF group. The detailed characteristic data were summarized Table 1.

### Surgical techniques

#### PE-PLIF

**Fig. 1:**
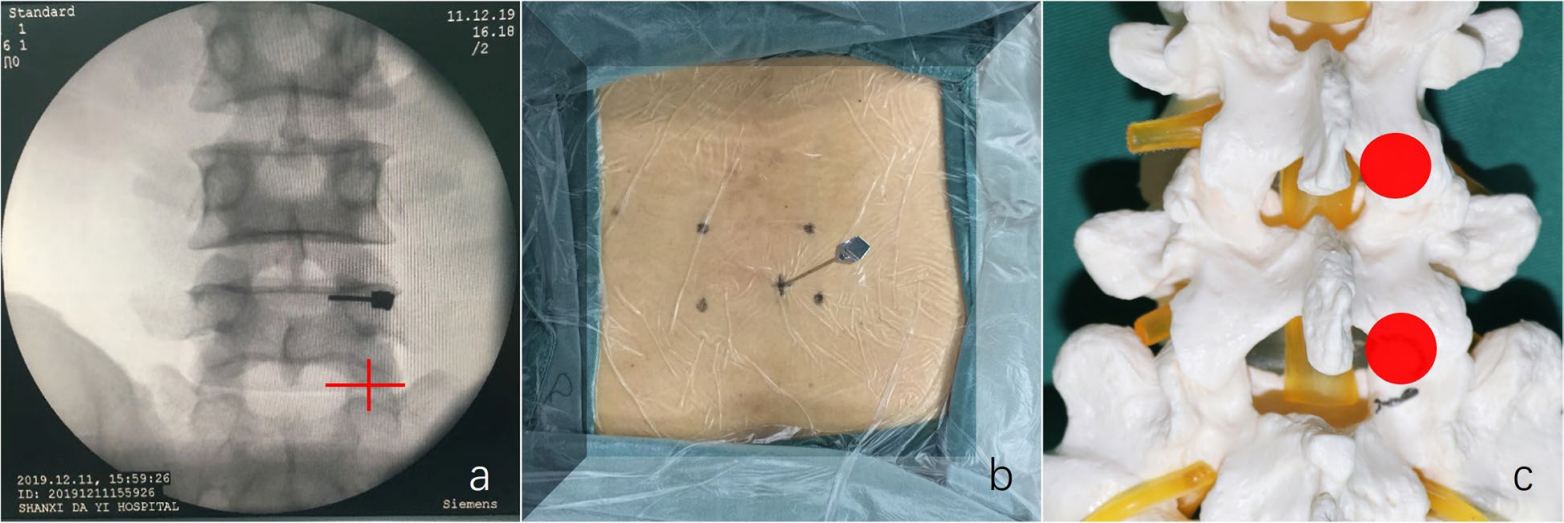
**a** The intersection of the red cross indicates the puncture site. **b** Location of the puncture needle. **c** Location and range of the articular process molding marked on the specimen

**Fig. 2:**
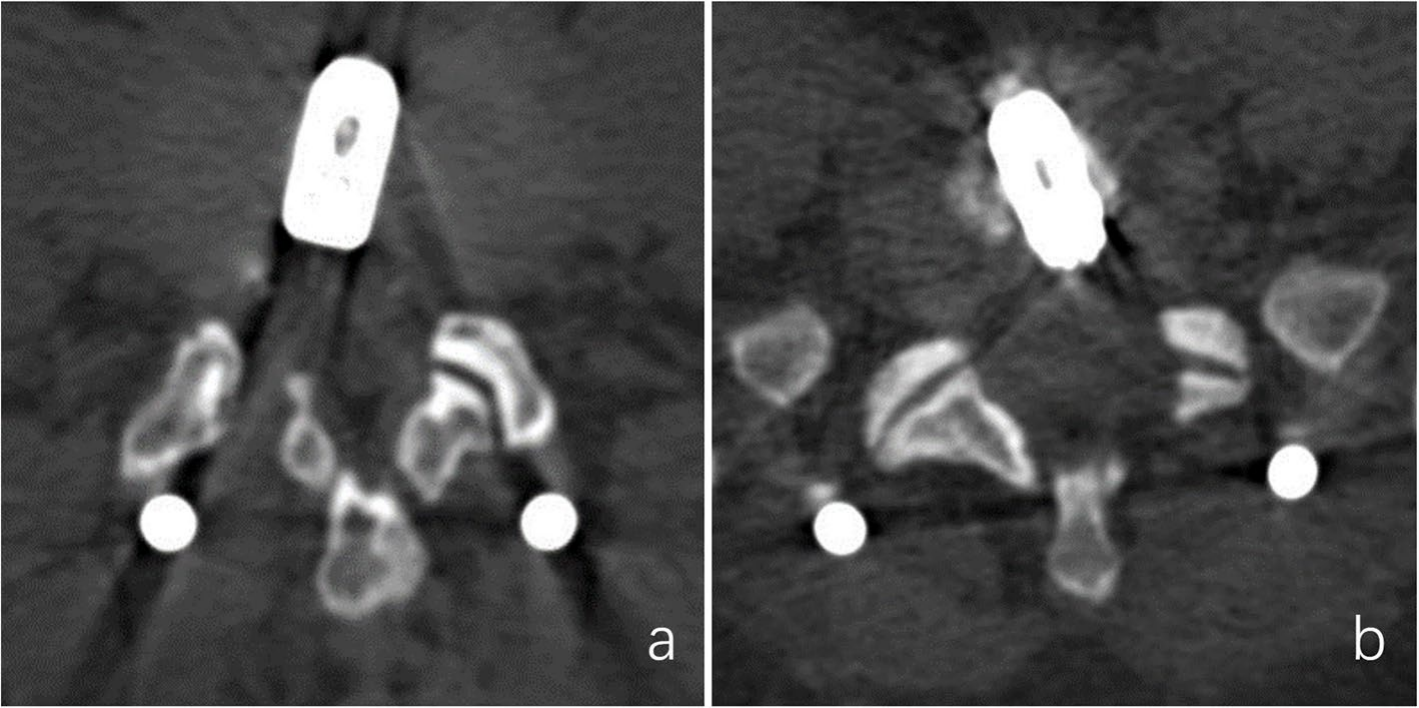
**a** Range of the articular process molding at lumbar 4–5. **b** Range of the articular process molding at lumbar 5-sacral 1

**Fig. 3:**
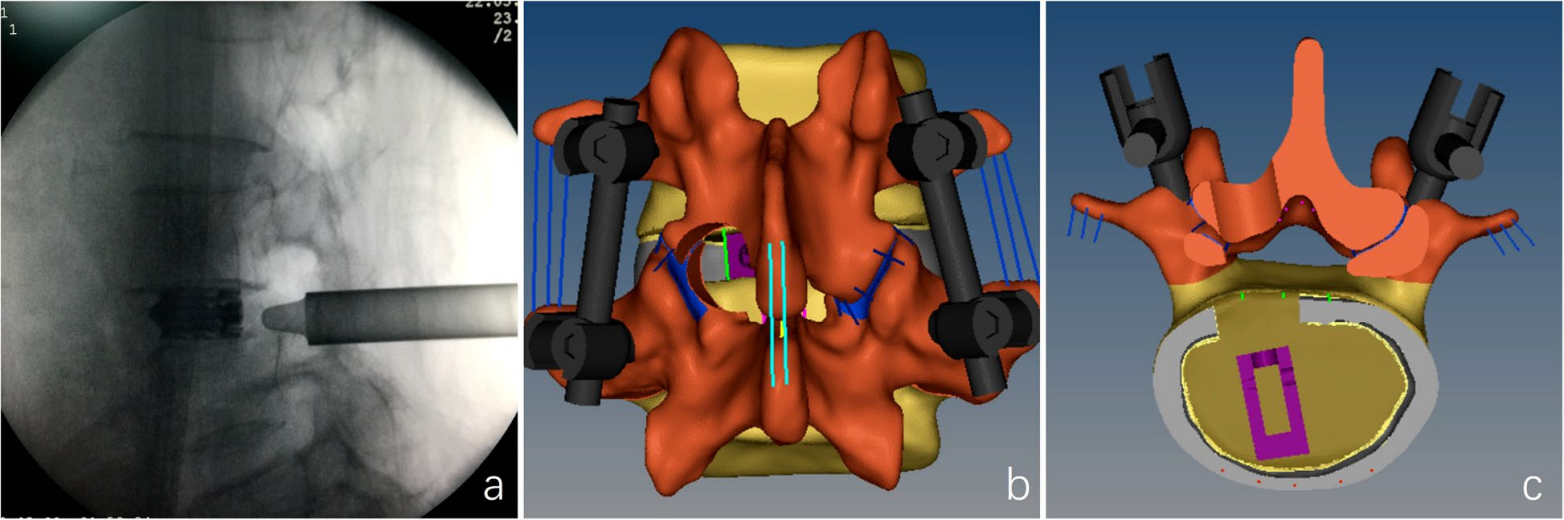
**a** Deployment of the cage to an appropriate height after it was placed via the working channel. **b** and **c** Schematic diagram of surgery

All operations were completed under general anesthesia. Patients were in the prone position. The table was adjusted to moderately flex the lumbar and expand the interlaminar space appropriately. On the anterior–posterior fluoroscopic view, the line parallel to the intervertebral space and the perpendicular through the midpoint of the articular process were marked. The intersection of these lines was the entry point, approximately 2 cm from the midline (Fig. 1). A longitudinal incision of approximately 13 mm was then made and gradually expanded with serial dilators to insert a working cannula (internal diameter: 11 mm). Next, an endoscope with a 10 mm outer diameter, a 7.1 mm working channel, and a 15° view angle (LUSTA endoscopic system, Spinendos, Germany) was inserted. The soft tissue was removed to expose the inferior articular process (IAP). An osteotome were used to resect the IAP and expose the superior articular process (SAP) below it. Next, the ligamentum flavum attachment at the medial SAP was separated, and a rongeur was used to remove the medial portion of the SAP to create ample space for the working cannula. We usually need remove the entire IAP at the L4-5 segment (Fig. 2a), and the medial two-thirds of the IAP at the L5-S1 segment (Fig. 2b). After the ligamentum flavum was partially removed, the nerve roots, dural sacs, and intervertebral discs were exposed. The working cannula was rotated so that its bevel moved toward the lateral side to prevent nerve roots from entering the working space. Nucleus forceps were used for discectomy, and an expandable reamer and a scraper were used to trim the cartilaginous endplate. Next, the endoscope was withdrawn, and a funnel-shaped bone graft delivery device was inserted into the working channel to fill the intervertebral space with shredded autogenous bone and osteoinductive material (bone morphogenetic protein-2). The delivery device was then removed, and an expandable cage (Beijing Ruizhi Tianhong Technology Co. Ltd., China) filled with autogenous bone and osteoinductive material was inserted and deployed to an appropriate height (9–13 mm) under C-arm fluoroscopic guidance (Fig. 3a). After endoscopic verification of decompression and the cage position, the endoscope and the working cannula were withdrawn. The table was returned to the neutral position, percutaneous pedicle screw fixation was performed on both sides. The Fig. 3b and c were schematic diagrams after all surgical procedures have been completed.

Open PLIF: After general anesthesia, the patient was placed in the prone position. An 8 cm incision was made along the midline. The paraspinal muscles were separated to expose the lamina and articular processes. Laminectomy decompression was performed, and the IAP and the medial portion of the SAP were removed to expose the dural sac and nerve roots. Then, a discectomy was performed. The same method as in the PE-PLIF was used to implant bone in the intervertebral space. A polyetheretherketone cage (length 22 mm, width 8 mm, height 7, 9, 11, or 13 mm) filled with autogenous bone and osteoinductive material was inserted after endplate preparation. Pedicle screw fixation was performed at the surgical segment.

### Clinical and imaging evaluations

The perioperative parameters, including operative time, blood loss, drainage amount, and complications, were reported. The outcome questionnaires were obtained when the patients were interviewed at the outpatient clinic. Visual analog scale (VAS) scores of low-back pain and lower limb pain were recorded before and after the operation at one week, one month, six months, twelve months, and the last follow-up postoperatively. The Oswestry Disability Index (ODI) score for functional status was recorded in the same fashion. The MacNab criteria were used to evaluate the satisfaction rate.

Lumbar lordotic angle (LLA) and segmental lordotic angle (SLA) were measured from plain standing radiographs. Fusion was determined by the Birdwell criteria according to computed tomography (CT) images at the last follow-up. The disc height (DH) was also evaluated, and cage subsidence was defined as a greater than 2 mm loss of height between the immediate and last postoperative images. The cross-sectional area of the spinal canal (CSAC) was compared on T2WI axial magnetic resonance images (MRI).

## Statistical analysis

Continuous variables such as age, VAS score, ODI score, SLA, LLA, and DH were displayed as the mean ± standard deviation and were analyzed with the independent sample t-test for intergroup comparisons and the paired t-test for intragroup comparisons. Nominal data such as complications, satisfaction rate, and fusion rate were analyzed with the χ^2^ test or Fisher’s exact test. P value of < 0.05 was considered indicative of statistical significance. Statistical analysis was performed using IBM SPSS, version 26.0 (IBM Corp., Armonk, NY, USA).

## Results

No significant difference was found at baseline characteristic data of age, gender ratio, BMI, smoker, diagnosis, mean follow-up between PE-PLIF and open PLIF groups (Table 1).

**Table 1:** Comparison of demographic data

	PE-PLIF (*n* = 30)	Open PLIF (*n* = 30)	P
Age (years)	51.6 ± 13.4	55.9 ± 14.3	0.232
Sex ratio (male/female)	18/12	18/12	1.000^*^
BMI	25.5 ± 3.3	25.1 ± 3.5	0.658
Smoker (yes/no)	14/16	8/22	0.108^*^
Segment (L4-5/L5-S1)	16/14	22/8	0.108^*^
Diagnosis			0.156^*^
Spondylolisthesis	3	7	
Instability	18	11	
Collapsed disc	9	12	
Mean follow-up (months)	24.7 ± 1.1	25.3 ± 1.5	0.121

*, results from fisher’s exact test or χ^2^ test. *BMI* Body mass index. *PE-PLIF* Percutaneous endoscopic posterior lumbar interbody fusion. *Open PLIF* Open posterior lumbar interbody fusion.

## Perioperative parameters

There was no conversion to open surgery in the PE-PLIF group. The operation time was 179.8 ± 31.9 min in the PE-PLI F group, which was significantly longer than that in the open PLIF group (125.8 ± 25.8 min; *P* < 0.05). Intraoperative blood loss was 63.3 ± 22.5 ml in the PE-PLI F group, which was significantly less than that in the open PLIF group (313.3 ± 183.1 ml; *P* < 0.05). Postoperative drainage was 614 ± 428.2 ml in the open PLIF group, whereas no postoperative drainage was required in the PE-PLIF group. The postoperative hospital stay was 3.3 ± 1.0 days in the PE-PLI F group, which was shorter than that in the open PLIF group (7.0 ± 1.0 days; *P* < 0.05). One patient in the PE-PLIF group developed contralateral neurological symptoms after the operation and did not respond to conservative treatment and thus underwent contralateral transforaminal endoscopic nucleotomy to alleviate the symptoms. One patient in the open PLIF group had cerebrospinal fluid leakage during the operation, which was resolved with conservative treatment. Another patient had a wound infection, which was resolved after secondary debridement and antibiotic treatment (Table 2).

**Table 2:** Comparison of intraoperative data and complications between the two groups

	PE-PLIF (*n* = 30)	Open PLIF (*n* = 30)	P
Operative times (min)	179.8 ± 31.9	125.8 ± 25.8	< 0.001
Estimated blood loss (ml)	63.3 ± 22.5	313.3 ± 183.1	< 0.001
Volume of drainage (ml)	NA	614 ± 428.2	NA
Postoperative hospitalization (days)	3.3 ± 1.0	7.0 ± 1.0	< 0.001
Complications (% n)	3.3	6.7	0.301^*^
Infection	0	1	
Dural tear	0	1	
Contralateral radiculopathy	1	0	
Reoperation	1	1	

^*^results from fisher’s exact test or χ^2^ test. *BMI* Body mass index. *PE-PLIF* Percutaneous endoscopic posterior lumbar interbody fusion. *Open PLIF* Open posterior lumbar interbody fusion

## Clinical efficacy

The VAS scores of low-back pain and leg pain and the ODI scores significantly improved postoperatively in both groups (*P* < 0.05). The VAS score of low-back pain was lower in the PE-PLIF group than in the open PLIF group at one week, three months, six months, twelve months postoperatively and the last follow-up (*P* < 0.05). The VAS score of leg pain was significantly lower in the PE-PLIF group one week, one month, and six months postoperatively (*P* < 0.05). There was no significant between-group difference in leg pain at the last follow-up. The ODI score was significantly lower in the PE-PLIF group at one month, three months, and six months postoperatively, but there was no significant between-group difference at twelve months and the last follow-up (*P* > 0.05). Based on the MacNab criteria, the overall satisfaction rate was 96.7% in both groups (Table 3).

**Table 3:** The clinical outcomes of the two groups

		PE-PLIF (*n* = 30)	Open PLIF (*n* = 30)	*p*
**VAS back pain**				
Preoperation		4.50 ± 1.23	4.23 ± 1.19	0.397
Postoperation	1 week	2.17 ± 0.69^*^	3.33 ± 0.65^*^	< 0.001
	1 month	1.77 ± 1.83^*^	2.10 ± 0.48^*^	0.339
	3 months	0.87 ± 0.63^*^	1.63 ± 0.72^*^	< 0.001
	6 months	0.67 ± 0.55^*^	1.13 ± 0.35^*^	< 0.001
	12 months	0.53 ± 0.51^*^	1.03 ± 0.49^*^	< 0.001
	Last	0.67 ± 0.48^*^	1.17 ± 0.38^*^	< 0.001
**VAS leg pain**				
Preoperation		6.17 ± 1.02	6.20 ± 0.96	0.897
Postoperation	1 week	2.17 ± 0.65^*^	2.53 ± 0.57^*^	0.024
	1 month	1.17 ± 0.38^*^	1.50 ± 0.51^*^	0.006
	3 months	1.00 ± 0.37^*^	1.07 ± 0.52^*^	0.570
	6 months	0.67 ± 0.58^*^	0.93 ± 0.45^*^	0.044
	12 months	0.60 ± 0.50^*^	0.63 ± 0.49^*^	0.795
	Last	0.53 ± 0.51^*^	0.60 ± 0.49^*^	0.610
ODI				
Preoperation		45.17 ± 4.96	45.90 ± 5.38	0.585
Postoperation	1 month	22.03 ± 3.33^*^	29.20 ± 4.99^*^	< 0.001
	3 month	15.10 ± 1.86^*^	20.97 ± 4.49^*^	< 0.001
	6 months	11.33 ± 2.38^*^	13.23 ± 3.46^*^	0.017
	12 months	9.97 ± 2.27^*^	9.90 ± 3.31^*^	0.928
	Last	10.1 ± 2.36^*^	9.73 ± 3.10^*^	0.576
Satisfaction rate (%)		96.7	96.7	1.000^**^
	Excellent	5	9	
	Good	24	20	
	Fair	1	1	
	Poor	0	0	

^*^, *P* < 0.05 compared to the preoperative data. **, results from fisher’s exact test. *VAS* Visual analog scale. *ODI*, Oswestry Disability Index

## Imaging findings

**Fig. 4:**
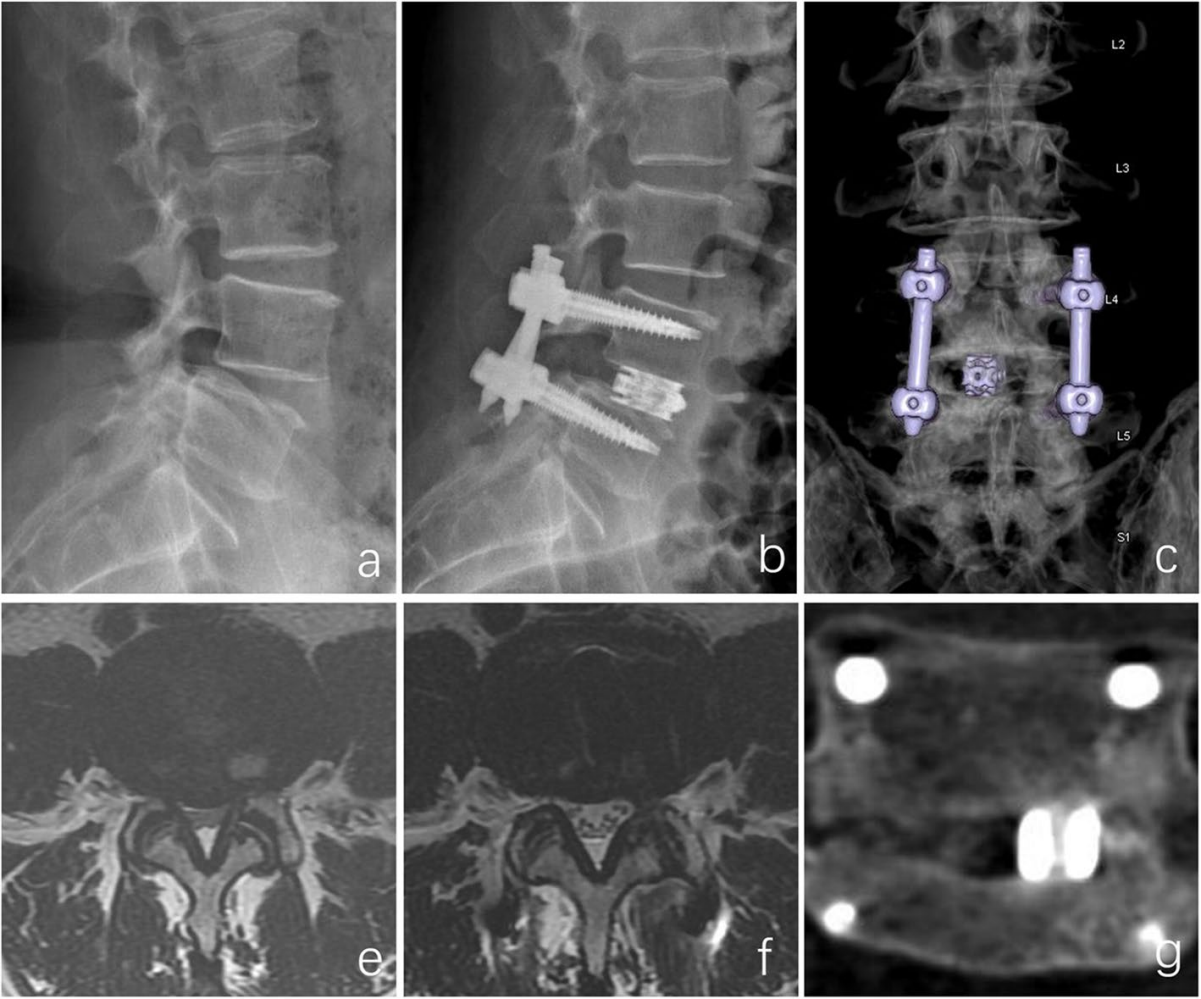
A 57-year-old woman complained of low-back pain and left lower limb pain and numbness. Preoperative lateral X-ray (**a**) shows spondylolisthesis (I°). Postoperative lateral X-ray (**b**) shows complete resolution of the spondylolisthesis and normal disc height. Reconstructed CT image (**c**) shows the cage position and the range of articular process molding. Axial MRI (**e, f**) shows significant improvement in the spinal canal and complete nerve decompression. Coronal CT (**g**) at the last follow-up shows definite interbody fusion

LLA, SLA, DH, and CSAC were significantly improved in both groups (*P* < 0.05), with no significant between-group difference (*P* > 0.05). Based on the Birdwell criteria, the fusion rate was 93.3% in the PE-PLIF group and 96.7% in the open PLIF group (*P* > 0.05). All unsuccessful cases were Birdwell category II, with no apparent translucent area around the cage or instrument failure. The cage subsidence rate was 6.7% in the PE-PLIF group and 16.7% in the open PLIF group (*P* > 0.05) (Table 4). An illustrative patient is shown in Fig. 4.

**Table 4:** The radiographic outcomes in the MI-PLIF and open PLIF groups

		PE-PLIF	Open PLIF	*P*
LLA (°)	Preoperation	33.30 ± 11.45	36.73 ± 6.29	0.156
	Postoperation	37.93 ± 7.13^*^	39.90 ± 5.11^*^	0.225
SLA (°)	Preoperation	14.17 ± 5.60	15.70 ± 3.83	0.228
	Postoperation	17.10 ± 4.55^*^	17.90 ± 3.33^*^	0.445
DH (mm)	Preoperation	9.21 ± 2.15	9.07 ± 1.72	0.771
	Postoperation	11.73 ± 1.27^*^	11.76 ± 1.33^*^	0.149
	Last follow-up	10.75 ± 1.18^*^	10.82 ± 0.98^*^	0.813
CSAC	Preoperation	0.92 ± 0.23	0.95 ± 0.23	0.638
	Last follow-up	1.70 ± 0.28^*^	1.66 ± 0.26^*^	0.556
Fusion rate (%)		93.3	96.7	1.000^**^
	Definite fusion	28	29	
	Probable fusion	2	1	
	Nonunion	0	0	
Cage subsidence		2	5	0.228^**^

^*^, *P* < 0.05 compared to the preoperative data. **, results from fisher’s exact test. *LLA* Lumbar lordotic angle. *SLA* Segmental lordotic angle. *DH*, Disc height. *CSAC* Cross-sectional area of the spinal canal

## Discussion

Minimally invasive spine surgery has been the trend in treating various degenerative spinal diseases. As an alternative, endoscopic spine surgery has attracted more attention in recent years. The previous literature regarding endoscopic spine surgery mainly emphasized preserving stability during the decompression of the neural structure. With the evolution of endoscopic instruments and techniques, the indications have been expanded with Endo-LIF and endoscope-assisted fusion. However, there is a lack of evidence to demonstrate the pros and cons of Endo-LIF. In the current study, the authors showed that the clinical and radiological outcomes in PE-PLIF could be comparable to those in the open PLIF group within two years of follow-up. In addition, PE-PLIF provided significant benefits in back and leg pain during the first six months postoperatively. Although PE-PLIF took a longer operative time than the open approach, patients undergoing PE-PLIF had less blood loss and shorter hospital stays. Our results indicated that PE-PLIF could be an alternative to open lumbar interbody fusion.

Currently, there are two main Endo-LIF techniques. In the transforaminal approach, the lateral and ventral SAP is partially removed to establish a working space. Early reports showed that the widened corridor allowed approximately 7 mm of working channel to dock on the target disc [11, 12]. In recent reports [13–15], surgeons can use a larger diameter working channel (outer diameter: 13–20 mm) to facilitate intraoperative decompression and cage placement by completely removing the SAP. The other technique is unilateral biportal endoscopic transforaminal LIF [16, 17]. The biportal endoscopic approach uses two incisions and channels (one for the endoscope and the other for the instrument) on the ipsilateral side for laminotomy and partial facetectomy. Unlike PE-TLIF, the surgical corridor of PE-PLIF is established by removing the IAP and the medial portion of the SAP, so the working area is in the lateral recess and subarticular zone. The advantages of PE-PLIF include 1) thorough decompression of the lateral recess and central canal by removal of the hypertrophic ligamentum flavum; 2) most spine surgeons are familiar with the posterior approach and easily overcome the learning curve; 3) a shorter working channel makes it easier to control instruments, which improves the efficiency of decompression and shortens the operative time; 4) endoscopic osteotomy is safer than trepan in PE-TLIF; and 5) a nucleus pulposus that prolapses upward or downward can be removed by expanding the working area accordingly.

This study showed that the operation time was significantly longer in the PE-PLIF group than in the open PLIF group. The reason might be that the endoscopic operation is inherently less efficient than open surgery due to using smaller instruments during facetectomy and discectomy. The mean operative time of PE-TLIF ranged from 167.5 to 285.7 min in previous reports [13, 14]. The surgeons’ experience and the instruments used may cause time variation between studies. In addition, the different trajectories may also affect the operative time. In theory, PE-PLIF takes a longer operative time than PE-TLIF due to medial facetectomy and central decompression. However, the mean operative time in the current study was similar to that of PE-TLIF performed by an experienced hand. In our opinion, the use of endoscopic osteotome rather than drilling may improve medial facetectomy efficiency. Blood loss was significantly lower in the PE-PLIF group than in the open PLIF group, resulting from smaller-scale bony resection, less muscular destruction, continuous intraoperative irrigation, and radiofrequency hemostasis during PE-PLIF. The features of perioperative parameters were similar to previous studies about minimally invasive spinal fusion by different techniques [16–18].

The current study showed that PE-PLIF was as effective as open PLIF but enhanced recovery after the operation. These findings may indicate that PE-PLIF could be a reliable alternative for minimally invasive lumbar fusion. Compared with open PLIF, PE-PLIF minimized damage to paraspinal muscles and significantly decreased iatrogenic low-back pain. In the study, only the VAS score of low-back pain at one month postoperatively had no significant between-group difference. The possible reason is that most patients undergoing open PLIF did not resume normal daily activities at that time. The low-back pain VAS score was significantly higher than that of the PE-PLIF group once daily activities resumed.

The first and most crucial radiological outcome in lumbar interbody fusion surgery is the fusion rate. In addition to techniques, implants and biologics should also be taken into consideration. The authors used static cages in the open PLIF but expandable cages in PE-PLIF. Static cages have been demonstrated to be reliable devices with a fusion rate of over 90% in previous studies [19, 20]. However, the common size of a static lumbar cage is not designed for Endo-LIF. Percutaneous transforaminal Endo-LIF has been reported as an alternative in some case series [13, 21]. Folman et al. [22] conducted a clinical study on an expandable cage in 2003 and showed that pseudoarthrosis occurred in only one of 87 patients, with no mechanical failure. In Lee et al. [12], a B-twin expandable cage was used to restore disc height; all 18 patients were followed up for an average of 46 months, 16 of whom achieved interbody fusion. Therefore, the authors adopted expandable cages in PE-PLIF to decrease the difference in the size and surface of cages between Endo-LIF and the open method. The radiological outcomes in the current study also showed comparable results [20].

Previous studies have shown that segmental lordosis and cage subsidence are related to functional outcomes [23, 24]. The incidence of cage subsidence was variable. Pisano et al. [25] reported a cage subsidence rate of 50.6% (mean: 5.5 mm) after TLIF. Lin et al. [19] reported a cage subsidence rate of 29.4% after MIS-TLIF and 25.0% after open PLIF. These variations may result from the measurement method (CT or X-ray), follow-up time, and cage material; therefore, no definitive conclusion can be drawn about the cage subsidence rate after lumbar fusion. In this study, the cage subsidence rate was 6.7% in the PE-PLIF group and 16.7% in the open PLIF group. Our results indicate that the use of an expandable cage may help prevent excess expansion of the intervertebral space and the resulting excess stress to the endplates, thereby reducing the cage subsidence rate, although further research is needed to test this hypothesis.

This study has some limitations. This was a nonrandomized study, which carries a risk of selection bias, although we strictly followed the inclusion and exclusion criteria during case selection. The sample size was small and, thus, failed to show a difference in the complication rate. Interobserver bias in the measurement of the radiological parameters may have been present. In addition, the follow-up time was less than two years in some petients.

## Conclusion

PE-PLIF is a safe and effective alternative for minimally invasive lumbar interbody fusion. Although the learning curve of PE-PLIF remains challenging and reflects a longer operative time, benefits such as enhanced recovery after surgery are significant and fewer complications are associated with less surgical trauma. With improvements in techniques and technologies, we believe that Endo-LIF will be widely applicable in the near future. Meanwhile, more well-designed studies are necessary to evaluate the benefit and cost-effectiveness of PE-PLIF.

## Data Availability

The dataset supporting the conclusions of this article is included within the article and its additional file.

## Abbreviations

PE-PLIF: Percutaneous endoscopic posterior lumbar interbody fusion
PLIF: Posterior lumbar interbody fusion
VAS: Visual analog scale
ODI: Oswestry Disability Index
Endo-LIF: Endoscopic lumbar interbody fusion
IAP: Inferior articular process
SAP: Superior articular process
LLA: Lumbar lordotic angle
SLA: Segmental lordotic angle
CT: Computed tomography
DH: Disc height
CSAC: Cross-sectional area of the spinal canal
MRI: Magnetic resonance imaging
